# Microplasma-Mediated Enhancement of FD-150 Uptake in HL-60 Cells

**DOI:** 10.3390/membranes15050156

**Published:** 2025-05-18

**Authors:** Mahedi Hasan, Jaroslav Kristof, Abubakar Hamza Sadiq, Md Jahangir Alam, Sadia Afrin Rimi, Farhana Begum, Kazuo Shimizu

**Affiliations:** 1 Graduate School of Science and Technology, Shizuoka University, Hamamatsu 432-8561, Shizuoka, Japan; h.s.abubakar.22@shizuoka.ac.jp (A.H.S.); s.a.rimi.21@shizuoka.ac.jp (S.A.R.); 2 Graduate School of Medical Photonics, Shizuoka University, Hamamatsu 432-8561, Shizuoka, Japan; kristof.jaroslav@shizuoka.ac.jp (J.K.); alam.md.jahangir.20@shizuoka.ac.jp (M.J.A.); f.begum.24@shizuoka.ac.jp (F.B.)

**Keywords:** microplasma, HL-60 cells, FD-150, transmembrane depolarization, membrane lipid order

## Abstract

Lipids are the primary components of cell membranes, and their properties can be temporarily modified by microplasma-generated species to enhance drug uptake. The ability of microplasmas to influence membrane dynamics has made them effective tools for facilitating drug uptake into cells. Despite this, the effect of microplasma irradiation on cell membranes is yet to be investigated. We investigated the effects of microplasma irradiation on fluorescein isothiocyanate-dextran 150 (FD-150) uptake in Human Promyelocytic Leukemia (HL-60) cells, with the focus on transmembrane potential and lipid order changes. Plasma was applied to HL-60 cells for five, seven, and ten minutes. Fluorescence intensity measurements showed that an uptake of FD-150 increased with treatment time, before declining at ten minutes of treatment. Following treatment, transmembrane potential analysis indicated transient hyperpolarization followed by gradual depolarization until 60 min, corresponding to increased FD-150 absorption. Analysis of the lipid order showed a more disordered membrane state, with the most pronounced changes observed at ten minutes. The increase in lipid disorder increases membrane permeability while excessive disruption of the lipid order impairs cell viability. These findings demonstrate the potential of plasma-generated reactive species in modulating membrane characteristics for intracellular drug delivery.

## 1. Introduction

Effective drug delivery remains a central challenge in medical research, particularly when aiming to maximize therapeutic efficacy while minimizing systemic toxicity. Conventional chemotherapeutics, for instance, lack cellular specificity and thus target all rapidly dividing cells, affecting healthy tissues such as those in the nails and hair follicles [1]. Due to their non-specific targeting, these treatments require drug doses that exceed safe levels for patients [2]. Additionally, delivering larger molecules such as antibodies presents another challenge in the treatment of diseases like cancer and other conditions. Over the years, various delivery systems—including hydrogels, liposomes, and nanoparticles—have been explored to overcome these limitations [3].

In parallel, biological delivery strategies using autologous or allogeneic cells [4], monoclonal antibodies [5], and genetic engineering cells [6] have been gaining attraction, especially for applications in cell therapy, tissue engineering, and oncology. Among the cell-based approaches, leveraging native blood cells—including leukocytes, erythrocytes, platelets, and stem cells—as delivery vehicles has shown significant promise [7,8]. Due to their ability to transendothelial migration [9], white blood cells such as HL-60 cells can be introduced into the bloodstream, making them promising candidates for use as delivery vehicles. White blood cells are also capable of delivering therapeutics to specific sites of infection, inflammation, or disease. Clinically, blood can be drawn from the patient, the desired cells isolated and cultured, and drug absorption can be achieved by in vitro microplasma treatment. Blood cells with a specific drug can then be reintroduced into that patient for a particular treatment.

Cellular internalization of drugs can be achieved either through surface binding or by facilitating intracellular uptake, both of which depend on membrane curvature, tension, and protein-mediated vesicle formation [10]. The adhesion of nanoparticles to lipid bilayers is dependent on parameters like adhesion energy density, nanoparticle diameter, and aspect ratio, and this can have a significant impact on drug delivery through membranes [11]. However, the bending energy required to form a vesicle is significantly higher than the interaction energy between proteins or their binding strength to membranes [12]. Thus, one of the key challenges in drug delivery is ensuring efficient cellular uptake. Several techniques have been developed to address this, including microinjection [13], viral vectors [14], nanoparticles, laser-assisted methods [15], electroporation [16], iontophoresis [16], and chemical-based approaches. Plasma discharge presents a distinct advantage by integrating multiple mechanisms that enhance drug uptake. It combines electrical currents, similar to iontophoresis, with high voltage effects observed in electroporation, while also utilizing reactive oxygen and nitrogen species to modulate cellular processes and promote membrane permeability [17,18].

Cold atmospheric plasmas produce reactive oxygen and nitrogen species (RONS), which can cause oxidation of lipids, creating pores in the membrane that enhance permeability [19,20]. Both large and permanent pores can disrupt the cell’s homeostasis and be lethal to the cell. Pores generated by plasma are temporary and limited in size. Plasma, through its iontophoresis effect [21,22], facilitates drug delivery into cells via endocytosis without causing damage to the cell membrane [23]. The effectiveness of drug delivery and the viability of cells are significantly influenced by the choice of plasma source. Effective drug delivery requires discharges where electric current passes through the cells. The combination of electrical effects and factors such as reactive oxygen and nitrogen species (RONS) produced by plasma irradiation results in a synergistic interaction, incorporating elements of iontophoresis and reactive species actions. This is facilitated by the electrodes in the cell solution [6] and the counter electrode of the micro-discharge system [4]. By modifying cellular physiology, cellular uptake of hydrophilic molecules can be improved through electrical stimulation and facilitate their delivery to the cytoplasm [7].

In melanoma cells, Dolezalova et al. [24] showed that very short plasma treatments were able to deliver plasmid DNA without affecting the viability of the cells. Studies have shown that the fluidity and permeability of lipid membranes—key factors in molecular transport—are directly influenced by lipid oxidation [25,26,27,28]. For instance, lipid peroxidation was shown to enhance nanoparticle endocytosis in gold nanoparticle-treated cells [29]. Moreover, membrane order influences permeability, with disordered (Ld) membranes being more permeable to water and other molecules compared to ordered (Lo) domains [30]. Transmembrane potential is also an important biophysical cue that regulates nutrient uptake and drug transport via membrane proteins [31,32].

The involvement of plasma-derived species in promoting endocytosis in adherent cells [29] and triggering membrane oxidation in model membranes [30] has been reported; however, how plasma exposure influences transmembrane potential and lipid order changes remains poorly understood, especially in suspension cells like Human Promyelocytic Leukemia (HL-60). Most previous studies have focused on either model membranes or adherent epithelial cells, leaving a gap in understanding plasma–cell interactions in suspension cells such as HL-60.

In this study, we investigate how air microplasma irradiation affects Human Promyelocytic Leukemia (HL-60) cells by assessing the uptake of a high-molecular-weight fluorescent probe, fluorescein isothiocyanate-dextran 150 (FD-150, MW 150 kDa). Our aim is to achieve maximum FD-150 uptake and elucidate the uptake mechanism by evaluating plasma-induced nanopore formation, changes in transmembrane potential, and relative lipid order. By examining these interconnected parameters, we aim to establish a biophysical link between plasma-induced membrane modifications and intracellular delivery efficiency, ultimately contributing to the development of optimized plasma-based drug delivery strategies.

## 2. Materials and Methods

### 2.1. Cell Culture Medium Preparation

Roswell Park Memorial Institute (RPMI 1640) medium was used for culturing the cells; it was obtained from Nissui Pharmaceutical Co., Ltd. (Tokyo, Japan). The antibiotic mixture containing penicillin and streptomycin, L-glutamine solution, Na_2_CO_3_ powder, and recombinant human insulin solution were obtained from Fujifilm Wako Pure Chemical Corporation in Osaka, Japan. Fetal bovine serum (FBS), originating from Australia, was obtained from Serana Europe GmbH in Pessin, Germany.

The cell culture medium was prepared by combining RPMI powder (5.1 g) with distilled water (500 mL), FBS (50 mL), L-glutamine solution (5 mL), antibiotic solution (5 mL), insulin (250 µL), and a 10% sodium bicarbonate solution (12 mL).

### 2.2. Cell Culture

HL-60 cells were purchased from the Cell Materials Development Office of Riken (Tokyo, Japan). Cells were seeded on a 25 cm^2^ culture flask. A total of 4 mL of culture medium was added and the mixture was incubated at 37 °C, 5% CO_2_ in an incubator (E-22, As One). After every three days, the culture medium was removed, and a fresh medium was provided.

### 2.3. Preparation of Cells for FD-150 Treatment

FD-150 was purchased from TdB Labs (Uppsala, Sweden). Its excitation and emission of 493 nm and 518 nm, respectively, can be used in the evaluation of lipid membrane permeability [33]. FD-150 was dissolved in distilled water to achieve a concentration of 0.5 mM. Using an aspirator, the culture medium was taken out of the culture flask and transferred into two 15 mL Falcon tube. These tubes were centrifuged at 210× *g* for 120 s, and the resulting pellets were mixed with 7 mL of RPMI (containing 1% FBS instead of 10% FBS). FBS concentration was reduced because a study reported that when plasma irradiation was applied, FBS showed cytotoxicity and mutagenic effect on cells [34]. Subsequently, 0.5 mL of this mixture was placed in a 60 × 15 mm culture dish with 2.5 mL of fresh RPMI containing 1% FBS (untreated control). To the remaining mixture, 99 µL of FD-150 was added a tube, and 0.5 mL of the mixture was distributed into additional 60 × 15 mm culture dishes, each containing 2.5 mL of RPMI. All dishes were incubated at 37 °C with 5% CO_2_ for 15 min. Following this, microplasma treatment was administered to the cells for five, seven, and ten minutes, and they were further incubated at a stable 37 °C with 5% CO_2_ for 1 h using an E-22 (As One) incubator. Non-irradiated cells served as controls, maintained under identical conditions as the treated cells.

### 2.4. Microplasma Treatment

**Figure 1:**
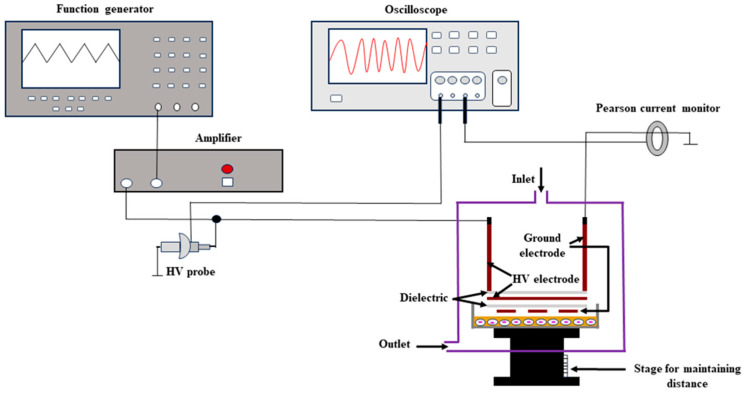
Schematic illustration of microplasma setup.

The experimental setup is similar to the earlier experiment with slight modifications [23] (Figure 1). The dielectric barrier discharge was produced using a thin-film electrode. A peak voltage of 4.1 kV and a frequency of 5 kHz were maintained for the air microplasma. Tektronix’s AFG3102 generated a saw-shape function of the positive voltage which was amplified by Trek’s 5/80 high-voltage amplifier. A dielectric barrier discharge was observed to have a typical discharge current. The electrode and medium distance could be adjusted in the treatment chamber for treating HL-60 cells. In the vacuum chamber, air did not flow; however, the inlet and outlet were open (open system), so the air was circulated between the inlet and the outlet. An electrode was placed 3 mm away from the cell medium, and microplasma irradiation was conducted for five, seven, and ten minutes.

### 2.5. Quantification of FD-150 Uptake

After 1 h of incubation at 37 °C in an incubator (As one), the cells were transferred to a 1.5 mL microcentrifuge tube and centrifuged at 300× *g* for 3 min at 25 °C. The supernatant was then removed using an aspirator, and the resulting pellet was resuspended in 1 mL of PBS. The cell suspension was subsequently centrifuged twice at 300× *g* for 3 min at 25 °C, and the pellet was resuspended in RPMI medium. Finally, 100 µL of cell suspension was transferred to a 96-well plate. The entire procedure was performed on a clean bench. Afterward, the fluorescence of FD-150 was evaluated in a microplate reader (Tecan, Zürich, Switzerland) at an excitation and emission wavelength of 493 and 518 nm, respectively.

### 2.6. Investigation of Transmembrane Potential

Bis-(1,3-Diethylthiobarbituric Acid) Trimethine Oxonol (DiSBAC_2_(3)) and Calcein Violet 450 AM were purchased from AAT Bioquest Inc.(Pleasanton, CA, USA) and eBioscience™ (ThermoFisher Scientific Inc., Waltham, MA, USA), respectively. Transmembrane potential was evaluated using the DiSBAC_2_(3) dye following the manufacturer protocol with slight modification. A 20 mM stock solution of DiSBAC_2_(3) and a 0.5% solution of Pluronic F-127 were prepared in DMSO. A total of 0.5 µL of DiSBAC_2_(3) was mixed with 0.5 µL of Pluronic F-127 and added to 3 mL of the culture suspension before plasma treatment. DiSBAC_2_(3) had an excitation wavelength of 530 nm and an emission wavelength of 560 nm. Additionally, Calcein Violet 450 AM was dissolved in DMSO to achieve a concentration of (1 mM) and added to the cell mixture at 1 µL per 3 mL to distinguish live cells, with excitation and emission wavelengths of 408 nm and 450 nm, respectively. Transmembrane potential was then evaluated at different time intervals using an Attune NxT Flow Cytometer (ThermoFisher Scientific, Waltham, MA, USA) equipped with four lasers. Measurements were taken using the yellow laser (Ex. 561 nm) with the YL1 channel (Em. 577–593 nm) and the violet laser (Ex. 405 nm) with the VL1 channel (Em. 415–465 nm). After 60 min of post irradiation, cell viability was evaluated according to Calcein Violet 450 AM staining.

### 2.7. Evaluating Membrane Lipid Order

Membrane lipid order was evaluated using the LipiORDER dye following the manufacturer protocol with slight modification. LipiORDER dye (Funakoshi Co. Ltd., Tokyo, Japan) was obtained in powdered form and dissolved in 100% dimethyl sulfoxide to prepare a 1 mM working solution. Precultured cells were centrifuged at 210× *g* for 120 s, and the resulting pellets were resuspended in RPMI. Subsequently, 0.5 mL of this suspension was transferred to a 60 × 15 mm culture dish containing 2.5 mL of fresh RPMI. LipiORDER (excitation: 405 nm; emission: 470–550 nm for the green channel and >550 nm for the red channel) dye was added to all dishes at a final concentration of 1 µL/mL. The cells then underwent microplasma treatment for five, seven, and ten minutes, while all other irradiation parameters remained consistent with previous descriptions. Following treatment, the cells were incubated at 37 °C with 5% CO_2_ for 1 h in an E-22 incubator (As One). Non-irradiated cells served as controls and were maintained under identical conditions. For flow cytometric analysis, cells were transferred into 1.5 mL centrifuge tubes, while microscopic observation was performed in 60 × 15 mm culture dishes.

After incubation, cells were analyzed using an Attune NxT Flow Cytometer (Thermo Fisher Scientific, Waltham, MA, USA) with a violet laser (Ex: 405 nm) and VL2 (Em: 500–525 nm), VL3 (Em: 575–627 nm) channels, as well as a microscope (Keyence BZ-X800) equipped with a green filter (Ex: 450–459 nm, Em: 500–550 nm) and a LipiORDER red filter (Ex: 405 nm, Em: 595–635 nm).

### 2.8. Statistical Analysis

Three autonomous repetitions were conducted for each examination. The significance of all data groups was statistically analyzed using the one-way ANOVA method, followed by post hoc and Duncan’s Multiple Range Test (DMRT), performed with SPSS Statistics 23 software. The graphs included in this report were created using GraphPad Prism 8.4.2. *, **, and *** indicate statistical significance at 5%, 1%, and 0.1% levels, respectively.

## 3. Results and Discussion

### 3.1. Microplasma Irradiation Induced FD-150 Uptake in Cells

**Figure 2:**
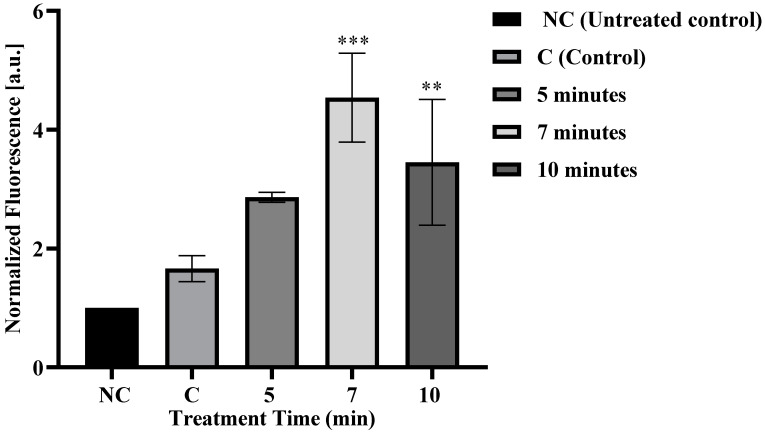
FD-150 fluorescence (with and without plasma treatment) after absorption by cells. Since cells exhibit intrinsic fluorescence, the fluorescence intensity for each condition was normalized to the fluorescence of the untreated control. The untreated control refers to cells maintained in the medium without FD-150 and microplasma treatment, while the control represents cells treated with FD-150 but without microplasma irradiation. Markers of 5 min, 7 min, and 10 min indicate cells treated with microplasma for five, seven, and ten minutes, respectively. ** *p* < 0.01 and *** *p* < 0.001 indicate statistical significance at the 1% and 0.1% levels, respectively.

The impact of treatment times on FD-150 absorption in HL-60 cells was examined. Cells were treated for five, seven, and ten minutes. Increasing treatment time showed an upward trend in the fluorescence intensity of FD-150 until seven minutes (Figure 2); afterward, it slightly declined. The fluorescence intensity of the samples treated for seven and ten minutes showed a significant difference compared to the control (Figure 2). The most effective absorption occurred at seven minutes of treatment time. This result is concurrent with our previous finding which revealed that plasma treatment enhanced FD-150 uptake [23,35]. In our previous study [35], plasma treatment at a 4 mm distance for 1, 3, and 5 min enhanced FD-150 uptake in HL-60 cells. The present study extends these findings by applying longer plasma exposures (5, 7, and 10 min) at a lesser distance (3 mm), allowing us to evaluate maximum FD-150 uptake and determine the optimal treatment duration for further mechanistic investigations. These results provide additional insight into the plasma-assisted delivery potential of FD-150. While the previous study in rat intestinal epithelial cells demonstrated that most FD-150 uptake occurred during microplasma treatment—with minimal or no effect from incubation or plasma-activated medium [23]—our current study in HL-60 cells showed that FD-150 uptake primarily occurred one hour after post irradiation. These contrasting results suggest that different cell lines may respond differently to microplasma treatment, and that drug uptake may occur either during treatment or during the incubation period depending on the cell type. Therefore, further comprehensive research across various cell lines and experimental conditions is necessary to elucidate the underlying mechanisms governing plasma-assisted drug uptake. Sasaki et al. [36] demonstrated that the continuous release of O^2•−^/ONOO^−^ leads to an increase in Ca^2+^ ions and enhanced YOYO-1 uptake, with these cellular responses being mediated by one or more TRP channels. Similarly, Jinno et al. [37] found that chemical particles do not independently facilitate drug delivery into cells; instead, their effectiveness is amplified through a synergistic interaction with electrical factors. Molecule size plays a crucial role in cellular uptake [38], with reactive oxygen species (ROS) influencing endocytosis for larger molecules. This suggests that microplasma could be an effective method for delivering large biomolecules, such as plasmid DNA into cells [39]. Our previous study demonstrated that FD-150 uptake significantly increases during the incubation period following plasma treatment [35], suggesting that long-lived reactive species, such as H_2_O_2_, play a crucial role in the uptake process. Consistently, the present study also observed substantial FD-150 absorption at one hour after plasma exposure. Kono et al. [40] demonstrated the cellular uptake of anionic liposomes in RAW264.7 cells after incubation with 100 or 200 μg lipid/mL for 3 h. Although cytotoxicity may occur at later stages, no significant reduction in cell viability was observed immediately following liposome uptake. Similarly, in our study, a significant increase in FD-150 uptake was observed in cells treated with microplasma for seven and ten minutes, followed by a one-hour post-irradiation incubation period. Notably, this increased uptake did not compromise cell viability. In contrast, Muller-Greven et al. [41] reported the internalization of bevacizumab into CD133^+^ cells after only 5 min of incubation. However, our approach offers greater potential, as it enables drug delivery through plasma-induced membrane modulation followed by incubation, providing an efficient method for enhancing cellular uptake.

Sakai et al. [42] successfully delivered 3.1 MDa DNA during plasma treatment, whereas Ikeda et al. [43] and Jinno et al. [44] reported the uptake of 2 MDa DNA into cells through indirect treatment. These variations indicate that cellular properties significantly influence molecular uptake mechanisms. Ikeda et al. [45] found that H_2_O_2_ concentrations up to 1 ppm facilitated molecular absorption by cells. Similarly, Cavalli et al. [46] demonstrated that treating cells with 50 μM H_2_O_2_ accelerated endocytosis by activating the p38 signaling pathway. Additionally, Sundqvist and Liu [47] confirmed that exposure cells to 0.1 μM H_2_O_2_ induced endocytosis in endothelial cells, further supporting the role of H_2_O_2_ in plasma-assisted molecular delivery. In our study, it is likely that long-lived reactive species generated by plasma contributed to FD-150 uptake in HL-60 cells. Moreover, our method offers a cost-effective alternative, relying only on electric current to produce reactive species—unlike some chemically synthesized agents such as singlet oxygen, which are both costly and short-lived [48].

### 3.2. Cell Viability

**Figure 3:**
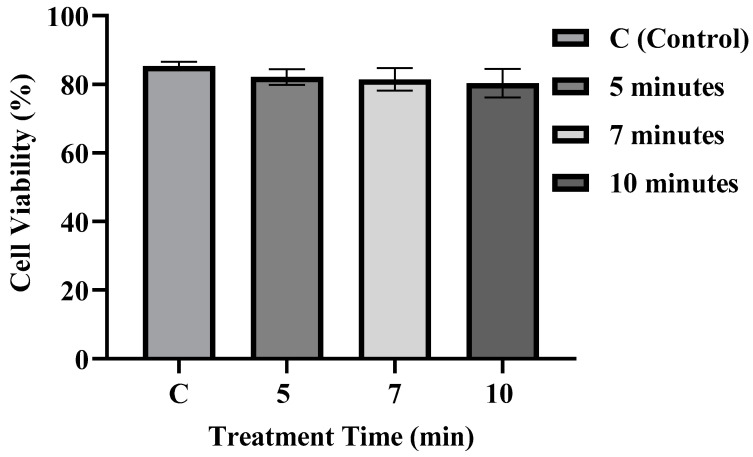
Cell viability of plasma-treated and nontreated samples at various treatment points. The histograms and gating strategy used for determining Calcein fluorescence are provided in Figure S1.

Calcein Violet 450 AM is a cell-permeable dye that is converted by intracellular esterases into a fluorescent, membrane-impermeable form, allowing selective staining of live cells. In contrast, apoptotic or dead cells with compromised membranes cannot retain the dye. Cell viability was around 85% in control. The percentage of live cells decreased slightly in microplasma-treated samples (Figure 3) compared to control; however, they are not statistically significant. Excessive concentrations of active molecules can diminish nutrient absorption into cells and potentially induce cell death. Biological cytotoxicity is often linked to a synergetic effect of H_2_O_2_, NO_2_, and other species such as ONOO^−^ [49]. These factors contributed to a slight reduction in cell viability. In plasma-treated cells, FD-150 uptake was significantly higher than in non-treated cells; however, optimizing plasma conditions is crucial for balancing delivery efficiency and cell viability. In this study, the seven-minute plasma treatment resulted in the highest FD-150 uptake while maintaining greater cell viability compared to other treatment durations, suggesting that seven minutes is the optimum condition among those tested.

### 3.3. Changes in Calcein Violet 450 AM Fluorescence and FD-150 Internalization in Cells

**Figure 4:**
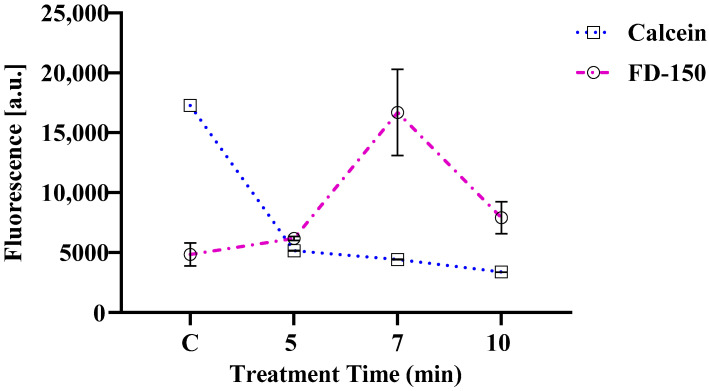
Changes in Calcein Violet 450 AM fluorescence and FD-150 internalization after 1 h incubation in control (C) and cells treated with plasma for 5, 7, and 10 min. The control (C) group includes cells exposed to FD-150 and Calcein Violet 450 AM without microplasma treatment. For better visual comparison with Calcein fluorescence, FD-150 fluorescence values are presented after multiplying by a factor of 10. Some error bars are not visible because they are shorter than the size of the symbol. As a result, Prism is unable to display them, even when the symbol is clear.

Calcein AM is a cell-permeable dye that enters cells without disrupting the plasma membrane. Inside metabolically active cells, intracellular esterases convert the non-fluorescent Calcein AM into fluorescent calcein, which is retained only in cells with intact membranes. Calcein Violet 450 AM is widely utilized for assessing cell viability and evaluating membrane integrity. Calcein can leak out of cells [50], indicating the presence of transmembrane pores, serving as a marker for compromised membrane integrity. Figure 4 shows changes in Calcein Violet 450 AM fluorescence and FD-150 internalization following plasma treatment for five, seven, and ten minutes compared to the untreated control after a 1 h post-irradiation incubation. Calcein fluorescence decreased progressively in plasma-treated cells, suggesting increased transmembrane damage and pore formation. In contrast, FD-150 fluorescence increased in treated cells, indicating enhanced intracellular uptake through these transmembrane pores. A similar nanopore formation mechanism induced by plasma-generated reactive oxygen species (ROS) was reported by Tero et al. [20] in artificial membranes. Additionally, Hasan et al. [21] demonstrated that electric stimulation can promote endocytosis-mediated siRNA uptake. In this study, FD-150 uptake peaked after seven minutes of plasma exposure but slightly decreased at ten minutes; therefore, the lack of correlation between decreasing Calcein fluorescence and FD-150 uptake indicates that mechanisms other than nanopore formation—such as endocytosis—may influence the internalization process. Similarly, Jinno et al. [38] observed that the transport mechanism differs between low-molecular-weight compounds like YOYO-1 and higher-molecular-weight molecules such as plasmid DNA. For larger molecules, reactive oxygen species (ROS) facilitate the uptake primarily by triggering endocytosis during plasma-mediated gene delivery.

### 3.4. Changes in Transmembrane Potential in Live Cells

DiSBAC_2_(3) is negatively charged dye (anionic pigments) used to study the transmembrane potential or polarization or charge of a membrane. Normally, the inside of a cell is negatively charged; when it decreases, it is depolarized (becoming less negative inside), and when it increases, it is hyperpolarized (becoming more negative inside). When a cell becomes more depolarized, it allows more DiSBAC_2_(3) to enter and binds with the membrane, causing its fluorescence to increase. The increased fluorescence indicates that more dye has entered, which reflects stronger depolarization. Similarly, FD-150 carries a negative charge at neutral pH due to the ionization of the fluorescein isothiocyanate (FITC) moiety conjugated to the dextran backbone [51], and its entry increases when the cell becomes more depolarized, like DiSBAC_2_(3).

**Figure 5:**
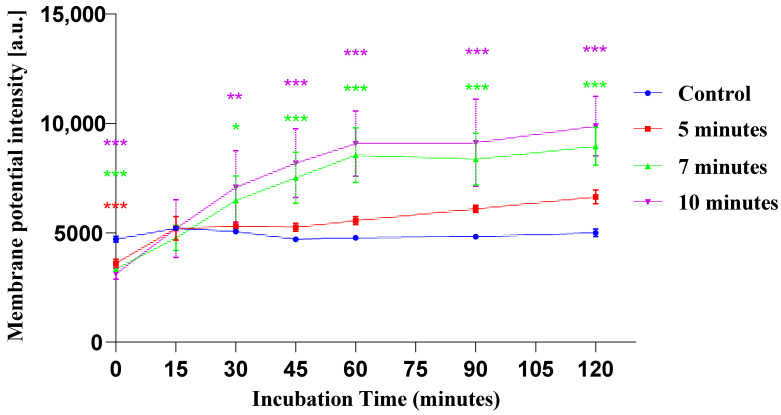
Changes in transmembrane potential at incubation period of 15, 30, 45, 60, 90 and 120 min for control (C), 5, 7, and 10 min for plasma-treated cells. Histograms and the gating strategy that was used for determining the transmembrane potential as well as calcein fluorescence are given in Figure S2. Each plasma-treated sample was compared to its respective control at each incubation time point (n = 5). *, **, and *** indicate statistical significance at 5%, 1%, and 0.1% levels, respectively. Different colored asterisks represent the corresponding treatments (red for 5 min, green for 7 min, and violet for 10 min of plasma treatment). Some error bars are not visible because they are shorter than the size of the symbol. As a result, Prism is unable to display them, even when the symbol is clear.

In Figure 5, plasma-treated samples show less fluorescence compared to the control immediately after treatment. This indicates hyperpolarization of the cell membrane. As the incubation time increased, the fluorescence of plasma-treated cells gradually increased until 60 min. Afterward, changes were minimal, and they remained almost constant. Although fluorescence remained stable beyond 60 min, seven- and ten-minute plasma-treated samples showed significantly higher fluorescence than their respective controls from 30 to 120 min post incubation. On the other hand, non-treated control showed almost constant fluorescence intensity throughout the incubation period, indicating no changes in transmembrane potential. At five minutes of cell plasma treatment, the fluorescence of DiSBAC_2_(3) was slightly more than that of the control from 30 min of post incubation. There was little difference between the transmembrane potential fluorescence of control and of cells plasma-treated for five minutes up to 60 min of post-irradiation incubation. Similarly, FD-150 absorption did not show significant changes for the five-minute treatment during 60 min of incubation. Seven-minute and ten-minute plasma treatments showed an upward trend of transmembrane potential fluorescence up to 60 min of incubation. Afterward, the changes were minimal, and the condition remained almost stable. Similarly, FD-150 uptake also significantly increased in seven- and ten-minute treatments for 60 min of incubation. Our result indicates that FD-150 uptake occurred within the 60 min of incubation during the depolarized state of the membrane. Among various plasmas, microplasma is characterized by its ability to induce an electric current within the culture medium surrounding the cells. This ion transport across the membrane can generate a current of approximately 1 nA, corresponding to a transmembrane potential in the range of 0.01–0.1 V. Jinno et al. [52] conducted a comparative study of different plasma sources and found that microplasma exhibited the highest gene transfection efficiency. In the melanoma cell line B16-F1, Mahadi et al. demonstrated that gene transfection can occur through macropinocytosis and caveolae-mediated endocytosis when a current of 0.34 mA/cm^2^ is applied for 15 min [21]. This electrical stimulation led to membrane depolarization, which is likely associated with the influx of calcium ions (Ca^2+^) and the activation of transient receptor potential (TRP) channels. The involvement of TRP and other cation channels may play a role in enhancing electric treatment (ET)-mediated molecular uptake [21]. Membrane depolarization is also known to promote proton-induced uptake, a process in which reduced extracellular pH triggers the formation of inward membrane invaginations and vesicles, thereby facilitating the internalization of macromolecules [53]. Similarly, our study demonstrate that microplasma-induced membrane depolarization significantly enhances the uptake of FD-150 in HL-60 cells, suggesting a similar underlying mechanism. In contrast, Lynda J.M. et al. reported that the influx of Ca^2+^ ions can activate calcium-activated potassium (BKCa) channels, resulting in membrane hyperpolarization. This shift in transmembrane potential may enhance the uptake of macromolecules by promoting mechanisms such as endocytosis and macropinocytosis [54].

Typically, molecules can be introduced into cells using plasma discharge through either direct or indirect treatment. Direct treatment occurs during the plasma application, resulting in a highly reactive environment with different plasma components including short-lived species in the liquid medium [55] that interact with the cells. On the other hand, the incubation period involves exposing the cells to long-lived molecules produced by the plasma after the initial discharge, making it less reactive than the direct approach. Vijayarangan et al. [56] showed that the “after-effect” of plasma treatment for drug uptake lasted for 30 min. This result showed that the effect of plasma treatment in changing the transmembrane potential remained until 60 min before becoming stable. This result also indicates that long-lived reactive species play a crucial role in the uptake processes, potentially by promoting endocytosis.

### 3.5. Changes in Membrane Lipid Order

**Figure 6:**
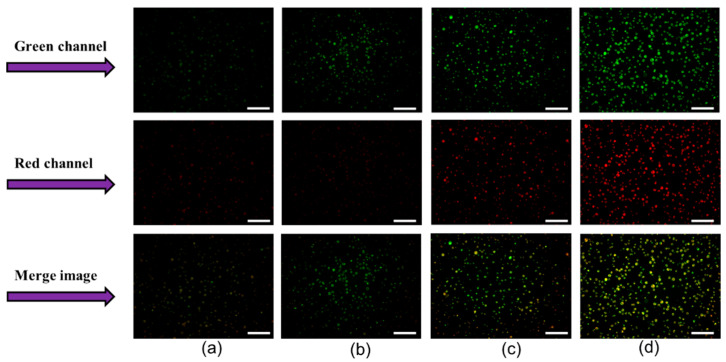
Membrane lipid order changes in HL-60 cells following microplasma irradiation. The top row represents the green channel, the middle row corresponds to the red channel, and the bottom row shows the merged images. Prior to irradiation, cells were stained with LipiORDER dye. (**a**) Control, (**b**) 5 min plasma-treated cells, (**c**) 7 min plasma-treated cells, and (**d**) 10 min plasma-treated cells. The green and red fluorescence indicate the liquid-ordered and liquid-disordered membrane phases, respectively. Images were captured at 20× magnification, with a scale bar of 20 µm.

Physical characteristics of the cell membrane are an important parameter for absorption of molecules into cells. These characteristics can be modified by microplasma, making it a promising tool for enhancing membrane permeability and drug uptake. Our study was conducted using a fluorescent solvatochromic pyrene probe (LipiORDER), which distinguishes liquid-ordered (Lo) from liquid-disordered (Ld) phases of membranes by polarity mapping [57]. Upon excitation at approximately 405 nm, the probe changes color from green (indicating the Lo phase) to red (indicating the Ld phase) depending on the order of the membrane lipids. Flow cytometric analysis revealed an increase in the fluorescence red (F_red_) to fluorescence green (F_green_) relative ratio in plasma-treated cells at one hour after irradiation (Table 1). The F_red_/F_green_ ratios of cells treated with plasma for five, seven, and ten minutes were all higher than that of the control group (Table 1). These findings were further validated through microscopic observation, where cell imaging demonstrated a gradual increase in both red and green fluorescence of plasma-treated cells (Figure 6a–d). Similarly, the merged images showed an enhanced orange or red coloration with increasing treatment time, indicating a shift in membrane lipids from an ordered (liquid-ordered) to a more disordered (liquid-disordered) phase (Figure 6a–d). The relative red-to-green fluorescence ratio more was measured using ImageJ 1.54g. All plasma-treated samples exhibited a significantly higher relative red/green fluorescence ratios than the control, confirming an increased membrane disorder with plasma exposure.

Both flow cytometric and microscopic analyses indicate that one hour after plasma irradiation, membrane lipid order shifts a more liquid-disordered (Ld) phase, enhancing membrane fluidity and facilitating FD-150 uptake. A similar trend was observed in intestinal cells after 4 min of post irradiation, as reported in [58]. However, while membrane order changes occurred earlier in intestinal cells, they were delayed in HL-60 cells. Although FD-150 uptake was highest in cells treated with plasma for seven minutes, transmembrane potential and lipid order changes were more pronounced in the ten-minute treatment group. A highly disordered (Ld) membrane phase impedes membrane recovery, as maintaining membrane integrity is crucial for cellular function. Cell viability slightly reduced in plasma treated cells indicating membrane order changes did not impede membrane recovery and cellular function.

Understanding which specific plasma discharge components influence membrane lipid order would provide valuable insights. Plasma generates both short-lived and long-lived reactive oxygen and nitrogen species (ROS and RNS) [59], which subsequently interact with the surrounding cell culture medium to form aqueous plasma-derived species. Additionally, plasma electrons can be converted into hydrated electrons in solution, leading to the transformation of dissolved oxygen into superoxide species. Through superoxide dismutation, these species are further converted into hydrogen peroxide (H_2_O_2_). Among the various ROS, H_2_O_2_ is particularly notable for its role in lipid oxidation, especially via the Fenton reaction, which produces hydroxyl (OH) and perhydroxyl (HO_2_) radicals. These highly reactive molecules can initiate lipid peroxidation in membrane phospholipids [60], thereby altering membrane fluidity, structural properties, and make it more permeable for drug uptake [61].

Previous studies have demonstrated that H_2_O_2_ concentrations up to 1 ppm can enhance molecular uptake into cells [45]. Additionally, recent findings indicate that plasma-derived long-lived species play a primary role in promoting FD-150 uptake [35]. Collectively, these studies indicate that plasma-generated species may contribute to modifications in membrane lipid order, a key factor in facilitating intracellular drug delivery. However, further investigation is required to determine whether microplasma-generated reactive species influence transmembrane potential, lipid organization, and drug uptake in HL-60 cells through endocytosis. A key aim of this study was to maximize FD-150 uptake and determine the optimal microplasma treatment conditions for future evaluation of gene expression related to endocytic pathways using RT-qPCR, along with assessment of the corresponding proteins.

## 4. Conclusions

In HL-60 cells, air microplasma treatment enhances the intracellular uptake of a 150 kDa fluorescent molecule (FD-150) by modulating membrane properties without compromising cell viability under optimized exposure conditions. FD-150 uptake increased with treatment duration, peaking at seven minutes of microplasma exposure followed by a one-hour post-treatment incubation. Beyond this duration, a decline in uptake was observed, suggesting that prolonged exposure may elicit counteracting effects that reduce transmembrane transport efficiency.

Notably, no direct correlation was observed between transmembrane pore formation and FD-150 internalization at longer treatment durations, indicating that nanopores are not the primary pathway for molecular entry. Instead, transmembrane depolarization and a shift in lipid phase from the ordered (Lo) to disordered (Ld) state were observed during the post-treatment phase, temporally coinciding with the increase in FD-150 uptake. These membrane alterations are likely mediated by long-lived reactive oxygen and nitrogen species (RONS), which enhance membrane fluidity and permeability. While nanopore formation may contribute to uptake, the discrepancy between pore presence and FD-150 internalization suggests that regulated mechanisms—such as endocytosis—may also be involved, although further validation is required.

This study advances our understanding of how plasma-induced membrane dynamics contribute to intracellular delivery and supports the potential of plasma-based strategies for enhancing targeted drug uptake in non-adherent cell systems. Future studies using the identified optimal treatment parameters should aim to clarify the underlying uptake mechanisms by evaluating the expression of key endocytosis-related genes via RT-qPCR, along with corresponding protein-level validation.

## Figures and Tables

**Table 1:** The relative ratios of red to green fluorescence in control and those subjected to five-, seven-, and ten-minute microplasma treatments. The observed intensity of the green (or red) channel is determined by the sensitivity of the detector for that channel and the properties of the optical elements used. This sensitivity differs for different colors and devices, as they use different detectors and optical components. Therefore, without proper calibration, direct quantitative comparison across instruments or settings is not feasible. Jurga et al. [57] addressed this issue by performing calibration using Labrafac oil (low polarity), large unilamellar vesicles (LUVs) composed of sphingomyelin (SM) and cholesterol (Chol) (intermediate polarity), and LUVs made from dioleoylphosphatidylcholine (DOPC) (higher fluidity and polarity) to enable quantitative assessment. In this study, such calibration was not performed; hence, the reported fluorescence ratios serve as qualitative indicators of whether membrane lipids are shifting toward a more ordered or disordered state. Histograms and gating strategies for the flow cytometry analysis are provided in Figure S3. Data are presented as mean ± error (n = 3, representing three independent replicates). ** *p* < 0.01, and *** *p* < 0.001 indicate statistical significance at 5%, 1%, and 0.1% levels, respectively.

Sample	F_red_/F_geen_ Ratio (Flow Cytometric)	F_red_/F_green_ Ratio (Microscopic)
Control	0.65 ± 0.01	0.78 ± 0.44
5 min	0.72 ± 0.02 ***	1.00 ± 0.15 **
7 min	0.70 ± 0.01 ***	1.13 ± 0.33 ***
10 min	0.80 ± 0.04 ***	1.34 ± 0.53 ***

## Data Availability

The original contributions presented in this study are included in the article/Supplementary Material. Further inquiries can be directed to the corresponding author(s).

## Supplementary Materials

The following supporting information can be downloaded at: https://www.mdpi.com/article/10.3390/membranes15050156/s1, Figure S1: Histograms and gating strategy for cell viability assessment using Calcein Violet 450 AM staining in plasma-treated and untreated samples at various treatment durations; Figure S2: Histograms and gating strategy for assessing changes in cell membrane potential using DisBAC_2_(3) and Calcein Violet 450 AM staining in plasma-treated and untreated samples at various treatment durations; Figure S3: Histograms and gating strategy for assessing changes in membrane lipid order using LipiORDER dye in plasma-treated and untreated samples at various treatment durations.
