# Supplementary material for: Microplasma-Mediated Enhancement of FD-150 Uptake in HL-60 Cells
Source: Membranes (Basel). 2025 May 18;15(5):156. doi: 10.3390/membranes15050156 (PMC12113648; doi:10.3390/membranes15050156)
Supplement: Supplementary file 1 [file membranes-15-00156-s001.zip › membranes-3531546-supplementary.pdf]

Cell viability:

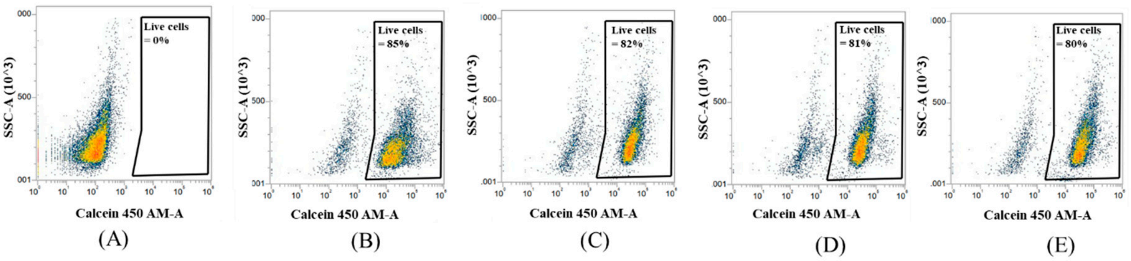

Figure S1: Histograms and gating strategy for cell viability assessment using Calcein Violet 450 AM staining in plasma-treated and untreated samples at various treatment durations.

Cell viability was analyzed after 60 minutes of post-irradiation in the following groups: (A) untreated control, (B) stained control, (C) 5-minute microplasma treatment, (D) 7-minute microplasma treatment, and (E) 10-minute microplasma treatment. The untreated control refers to cells maintained in culture medium without Calcein Violet 450 AM staining and microplasma treatment. The stained control group includes cells stained with Calcein Violet 450 AM but without microplasma irradiation. All values are presented as the average of three independent replicates.

Changes in membrane potential in live cells:

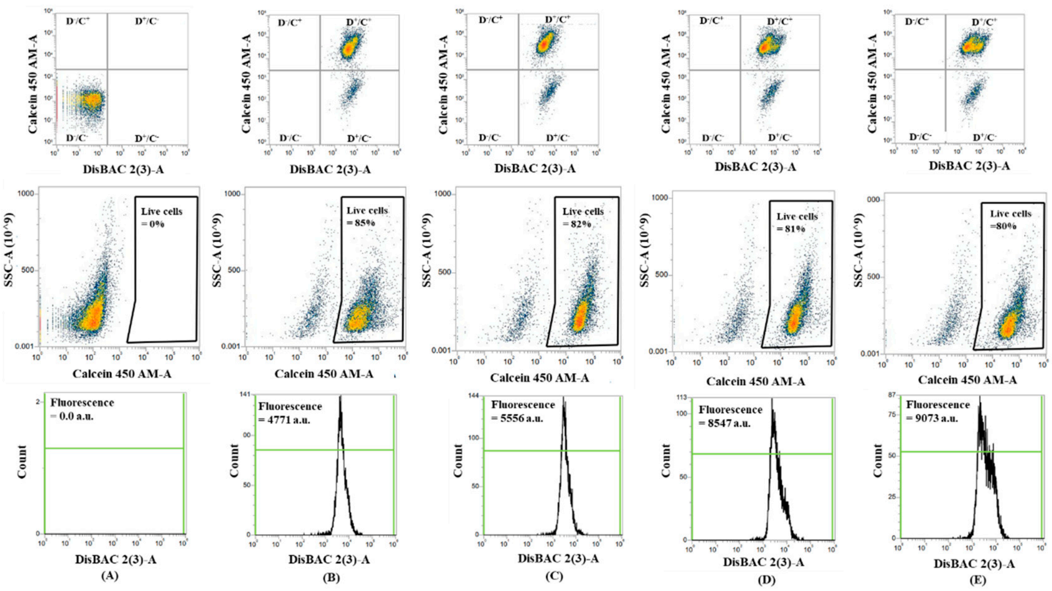

Figure S2: Histograms and gating strategy for assessing changes in cell membrane potential using DisBAC$_2$ (3) and Calcein Violet 450 AM staining in plasma-treated and untreated samples at various treatment durations.

Membrane potential changes were evaluated at incubation times of 0, 15, 30, 45, 60, 90, and 120 minutes for the following groups: (A) untreated control, (B) stained control, (C) 5-minute microplasma treatment, (D) 7-minute microplasma treatment, and (E) 10-minute microplasma treatment. The untreated control refers to cells maintained in culture medium without DisBAC$_2$ (3) and Calcein Violet 450 AM staining and microplasma treatment. The stained control group includes cells stained with both DisBAC$_2$ (3) and Calcein Violet 450 AM but without microplasma irradiation. The top row (quadrant plots) displays dual-stained cells with DisBAC$_2$ (3) and Calcein Violet 450 AM:

D^-/C^+: Cells stained with Calcein Violet 450 AM but not with DisBAC$_2$ (3)), indicating live cells without changes in membrane potential.

D^-/C^-: Cells negative for both stains, representing dead cells with no detectable membrane potential changes.

D^+/C^-: Cells stained with DisBAC$_2$ (3) but not with Calcein Violet 450 AM, indicating dead cells with altered membrane potential.

D^+/C^+: Cells positive for both stains, representing live cells exhibiting changes in membrane potential (desired population).

The middle row shows Calcein Violet 450 AM fluorescence, indicating the percentage of live cells in each condition.

The bottom row presents DisBAC$_2$ (3) fluorescence, reflecting membrane potential changes under each treatment condition compared to the control. All values are presented as the average of five independent replicates.

Changes in membrane lipid order:

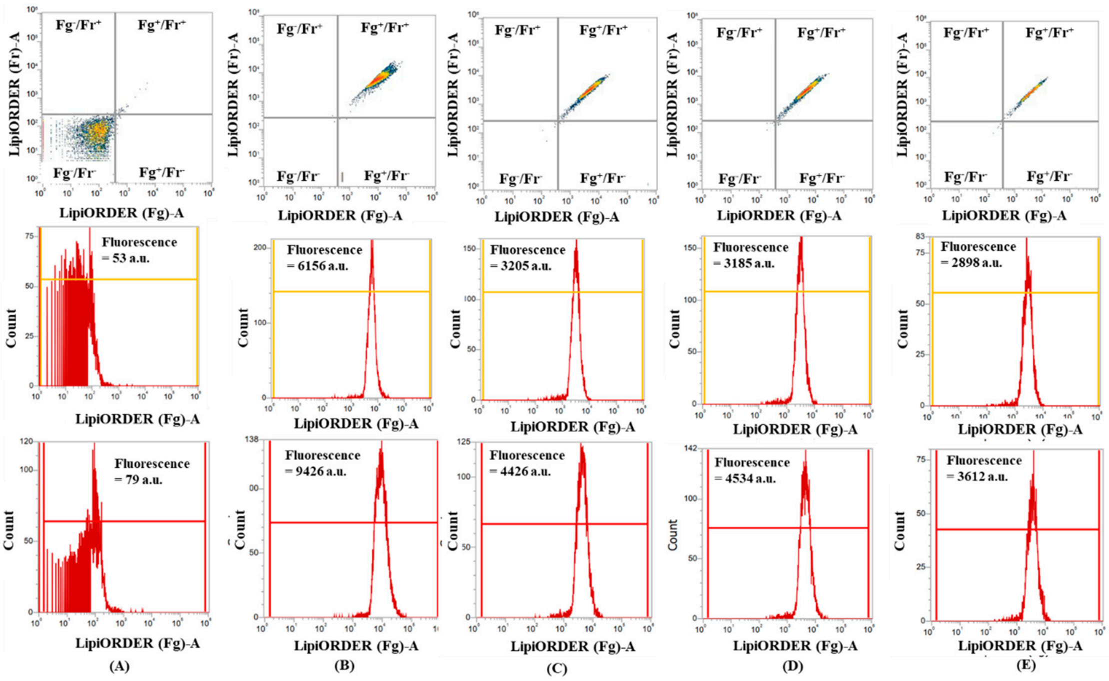

Figure S3: Histograms and gating strategy for assessing changes in membrane lipid order using LipiORDER dye in plasma-treated and untreated samples at various treatment durations.

Lipid order was evaluated after 1 hour of post-irradiation incubation in the following groups: (A) untreated control, (B) stained control, (C) 5-minute microplasma treatment, (D) 7-minute microplasma treatment, and (E) 10-minute microplasma treatment. The untreated control refers to cells maintained in culture medium without LipiORDER staining and microplasma treatment. The stained control group includes cells stained with LipiORDER but not exposed to microplasma irradiation.

The top row (quadrant plots) displays fluorescence signals in the red (Fr) and green (Fg) channels:

Fg^-/Fr^+: Cells showing red fluorescence but no green fluorescence, representing the liquid-disordered phase.

Fg^-/Fr^-: Cells showing neither green nor red fluorescence.

Fg^+/Fr^-: Cells showing green fluorescence (indicative of the liquid-ordered phase) but no red fluorescence.

Fg^+/Fr^+: Cells positive for both green and red fluorescence, representing a mixture of liquid-ordered and liquid-disordered phases. Changes in lipid order were determined by comparing the ratio of red to green fluorescence between the control and treated groups.

The middle row shows red fluorescence (Fr), indicating the extent of the liquid-disordered phase.

The bottom row presents green fluorescence (Fg), reflecting the extent of the liquid-ordered phase.

All values are presented as the average of three independent replicates.
